# Staff training in positive behaviour support for behaviour that challenges in people with intellectual disability: cost-utility analysis of a cluster randomised controlled trial

**DOI:** 10.1192/bjo.2020.1

**Published:** 2020-02-05

**Authors:** Rachael Maree Hunter, Victoria Vickerstaff, Michaela Poppe, Andre Strydom, Michael King, Ian Hall, Jason Crabtree, Rumana Omar, Vivien Cooper, Asit Biswas, Angela Hassiotis

**Affiliations:** Associate Professor, Research Department of Primary Care and Population Health, University College London, UK; Senior Research Fellow, PRIMENT Clinical Trials Unit, University College London, UK; Clinical Trial Manager, Division of Psychiatry, University College London, UK; Professor in Intellectual Disabilities, King's College London and South London and the Maudsley NHS Foundation Trust, UK; Professor of Primary Care Psychiatry, Division of Psychiatry, University College London, UK; Consultant Psychiatrist and Clinical Lead, Community Learning Disability Service, East London NHS Foundation Trust, UK; Clinical Psychologist, East London NHS Foundation Trust, UK; Professor of Medical Statistics, Department of Statistical Science, University College London, UK; Chief Executive, Challenging Behaviour Foundation, UK; Consultant Psychiatrist, Agnes Unit, Leicestershire Partnership NHS Trust & University of Leicester, UK; Professor of Psychiatry of Intellectual Disability, Division of Psychiatry, University College London; and Camden and Islington NHS Foundation Trust, UK

**Keywords:** Intellectual disability, randomized controlled trial, cost-effectiveness

## Abstract

**Background:**

Behaviour that challenges in people with intellectual disability is associated with higher healthcare, social care and societal costs. Although behavioural therapies are widely used, there is limited evidence regarding the cost and quality-adjusted life-years (QALYs).

**Aims:**

We aimed to assess the incremental cost per QALY gained of therapist training in positive behaviour support (PBS) and treatment as usual (TAU) compared with TAU using data from a cluster randomised controlled trial (Clinical Trials.gov registration: NCT01680276).

**Method:**

We conducted a cost-utility analysis (cost per QALY gained) of 23 teams randomised to PBS or TAU, with a total of 246 participants followed up over 36 months. The primary analysis was from a healthcare cost perspective with a secondary analysis from a societal cost perspective.

**Results:**

Over 36 months the intervention resulted in an additional 0.175 QALYs (discounted and adjusted 95% CI −0.068 to 0.418). The total cost of training in and delivery of PBS is £1598 per participant plus an additional cost of healthcare of £399 (discounted and adjusted 95% CI −603 to 1724). From a healthcare cost perspective there is an 85% probability that the intervention is cost-effective compared with TAU at a £30 000 willingness to pay for a QALY threshold.

**Conclusions:**

There was a high probability that training in PBS is cost-effective as the cost of training and delivery of PBS is balanced out by modest improvements in quality of life. However, staff training in PBS is not supported given we found no evidence for clinical effectiveness.

Between 10 and 15% of adults with intellectual disability display behaviours that challenge, with aggression the commonest type.^1^ Behaviours that challenge are associated with long-term hospital admission (often out-of-area), restrictive care practices and neglect as well as increased service use and prescription of antipsychotic medication.^2^ It is commonly cited that behaviours that challenge drive cost of care for people with intellectual disability, because of high staffing levels and long in-patient admissions,^3–6^ as well as contributing to family burden and reliance on family carers.^7^ Although previous studies have found significant associations between degree of intellectual disability, behaviours that challenge and service costs,^3,6,8^ these are based on small observational studies or localised examples of services that may not apply in other parts of the UK or having been superseded by advances in community health and social care.^9^

Recently, the National Institute for Health and Care Excellence (NICE) recommended that intellectual disability services in England deliver personalised interventions for behaviours that challenge within the framework of a complex intervention, namely PBS.^10^ PBS incorporates applied behavioural analysis with the aim of understanding the purpose of an individuals' behaviour and context in which it occurs followed by the development of a personalised support plan ostensibly leading to reduction in such behaviours and improvement in quality of life. Although most frontline health professionals would have had a basic understanding of behavioural techniques or even been aware of PBS, a subgroup would need to have acquired further skills in delivering manual-assisted PBS. As part of a cluster randomised controlled trial (RCT), we undertook a health economic evaluation of health staff (referred to as therapists) training in PBS.^11–13^

## Aims

We report the mean incremental cost per quality-adjusted life-year (QALY) gained of staff training in PBS and treatment as usual (TAU) compared with TAU only from a healthcare cost perspective in line with NICE guidance^14^ over 36 months to capture costs and outcomes over a longer time horizon. We also report the incremental cost per QALY gained at the end-point of 12 months, consistent with the primary end-point for clinical effectiveness, and from a wider societal cost perspective including the cost of supported accommodation, criminal justice costs and the impact on informal carers.

## Method

Twenty-three community intellectual disability services that support adults with intellectual disability who display behaviours that challenge took part in a multicentre, single-blind, two-arm, parallel cluster RCT^12^ between 2013 and 2016. Eleven services were randomised to the intervention and TAU arm and 12 to the TAU arm. In total, 246 adults across the range of intellectual disability were recruited to the study.

Participants were included in the study if they were 18 years or older, had a total of 15 or higher on the outcome measure at screening (Aberrant Behaviour Checklist-Community version (ABC-C);^15^ 15 or higher corresponding with a degree of challenging behaviour occurring at least weekly including verbal or physical aggression, hyperactivity, refusal to attend activities and non-responsiveness that requires professional input), had no acute mental illness or a personality disorder and the intellectual disability service agreed to participate. The primary objective of the trial was the clinical effectiveness of manual-assisted training in PBS in reducing behaviours that challenge, measured at baseline, 6 months and 12 months.

A naturalistic observational study was conducted from 12 months to 36 months to examine the longer-term impact of the intervention on clinical outcomes, costs and QALYs. Once all the participants in a service had completed their 12-month follow-up, they were not restricted in receiving further training in behavioural support or other interventions. The study (Clinical Trials.gov registration: NCT01680276) was approved by the National Research Ethics Service Committee London–Harrow (reference 12/LO/1378). Written consent was obtained from all participants. Further details on the trial can be found elsewhere.^11–13^

### Interventions

In services randomised to the intervention arm, therapists received manual-assisted face-to-face training in PBS by expert trainers in three 2-day workshops over 15 weeks.^11^ Post-training mentoring for at least 1 year was also provided, which mainly consisted of communication via email, monthly teleconferences and site visits by the research team.

Teams that were allocated to TAU only continued with existing treatment approaches, which employed a multiprofessional model to the management of behaviours that challenge. These included behavioural, psychosocial and pharmacological approaches, for example physical health checks, simple behavioural modification, other psychosocial interventions, such as cognitive–behavioural therapy and prescribing of psychotropic medication.

### Cost of PBS training and intervention

To calculate the cost of the intervention, data were collected on costs of training the therapists. This included time allocated to attend the training sessions, the cost of specialist and academic time to run the training sessions and mentoring, training materials and travel costs. As a conservative estimate we calculated the total cost per participant of training as the total cost of training divided by the number of participants in the intervention arm. Two to three therapists per intellectual disability service volunteered to train in and deliver PBS.

Therapists were asked to report the amount of time they spent delivering PBS. The tasks included conducting assessments, direct contact with participants and working with paid carers, families or participants to implement the support plan. The mean total hours of delivering PBS per participant were calculated and multiplied by the average cost per hour of an equivalent National Health Service (NHS) Agenda for Change Band 6 staff (experienced in assessment, diagnosis and treatment of mental disorders in in-patient or community settings).^16^

### Resource use and costs

Resource use was collected using a modified version of Client Service Receipt Inventory (CSRI) adapted for the study.^17^ The CSRI was completed by family or paid carers at baseline, 6, 12 and 36 months asking about the preceding 6 months on the number of primary and secondary health and social care contacts, type of housing, other carer input and criminal justice contacts.

Resource use was multiplied by unit costs to calculate the mean total cost per participant of each resource at baseline, 6, 12 and 36 months. Total cost of each contact was calculated as the hourly rate of face-to-face contact (based on Personal Social Services Research Unit^16^ costs) multiplied by the average duration of an appointment with that professional. Unit costs and sources for appointment duration are reported in supplementary Table 1 (available at https://doi.org/10.1192/bjo.2020.1). Medication costs were based on the British National Formulary.^18^

Unpaid carers (family and close others) often provide essential support and care to participants with intellectual disability.^19^ Their contribution needs to be recognised as they provide significant amounts of care for vulnerable individuals. As a result, we included the estimated cost of time spent by unpaid carers (family and significant others) caring for the participants in the study, at an hourly rate of £24,^16^ the same as for a home care worker.

Societal costs also include private service use or out-of-pocket expenses. Given that there are no nationally published sources specifically for the cost of private healthcare and there were missing data regarding out-of-pocket costs, these were priced at the same level as public healthcare costs. Type of accommodation was categorised as residential, supported living and independent living with floating support (the latter being a flexible service provided by external agencies to fairly able participants in order to maintain their independence). Costs for residential accommodation were based on the number of bedrooms in the property.^20^ The cost of supported living was categorised as ongoing support, for example 24 h care or less than 24 h. All costs are in 2014/2015 British pounds sterling.

### Outcome measure

Participants and family or paid carers both completed the EQ-5D Youth (EQ-5D-Y)^21^ at baseline, 6, 12 and 36 months. The EQ-5D-Y was chosen because of the simpler language making it easier for participants with intellectual disability to understand and complete. Utility scores to calculate QALYs were calculated from carer responses to the EQ-5D-Y at baseline, 6, 12 and 36 months and the EQ-5D 3 level (EQ-5D-3L) tariff formula.^22^

We conducted a sensitivity analysis using the responses from adults with intellectual disability. There is limited evidence on the validity of using the EQ-5D to calculate quality of life for people with intellectual disability. We developed a linear multi-level model of the the EQ-5D-Y proxy responses at each time-point compared to the ABC-C to test if the EQ-5D-Y as completed by proxies (families and paid carers) is sensitive to changes in primary outcome (ABC-C) and hence is valid in detecting changes in challenging behaviour.

### Statistical analysis

While the study was powered to detect a mean difference of 0.45 of a standard deviation (s.d.) in ABC-C between the two arms post-intervention, the primary clinical outcome for the RCT, it was not powered to detect differences in costs and utilities. In line with recommendations made elsewhere^23,24^ we take a probabilistic approach to aid decision-making for resource allocation, calculating the probability that staff training in PBS is cost-effective for a range of values of willingness to pay for a QALY gained. All analyses were based on intention to treat and correspond with the analyses in the published clinical effectiveness paper.^12^

Data were assumed to be missing at random and imputed using multiple imputations with chained equations.^25^ Variables identified as predictive of missingness (intellectual disability level, current living situation and accommodation type) were included in the imputation model. We imputed 35 data-sets, equal to the percentage of missing data (35%).

The mean incremental total cost of the intervention arm compared with control was calculated using regression analysis, adjusting for baseline costs and accounting for clustering by site as random effects. The 95% CIs for health and social care and societal costs were calculated using bias corrected bootstrapping with 7000 draws.^23^

QALYs were calculated from baseline, 6-, 12- and 36-month utility scores as the area under the curve. If a participant were to die during the study, they were entered as 0 at the date of death and QALYs calculated as a straight line from their last available measurement until the date of death. Mean incremental QALYs were calculated using regression analysis adjusting for baseline values^26^ and staff/participant ratios and with site as a random effect. The 95% CIs were calculated using bootstrapping with 7000 draws.^23^

Two-stage bootstrapping is generally the recommended method for calculating the incremental cost-effectiveness ratio in cluster RCTs as it accounts for the correlation between costs and QALYs. However, two-stage bootstrapping is not possible when covariate adjustment is required to account for baseline imbalances as the additional covariates cannot be included in the model. Instead, standard methods of bootstrapping are less likely to result in bias in these instances.^27^ Given that there were baseline imbalances between the two trial arms in costs and utilities, we used a standard linear regression to calculate the beta coefficient for the treatment effect, adjusting for baseline measures and including the size of the team as a covariate with clustering as a random effect. The beta coefficients for each arm for costs and QALYs were captured for each bootstrap and across the 35 imputed data-sets for 200 replications per bootstrap.^28^

The bootstrap beta coefficients generated a cost-effectiveness acceptability curve for a range of values of willingness to pay for a QALY gained.^24^ Cost-effectiveness planes are also reported. The probability that the intervention is cost-effective compared with TAU at a willingness to pay of £30 000 per QALY gained is reported in line with NICE guidance.^14^  Costs and QALYs after 12 months were discounted at a rate of 3.5% in line with NICE guidance for the 36-month values.^14^

The sensitivity of the EQ-5D-Y completed by family and paid carers to changes in challenging behaviour was assessed using a multilevel model looking at EQ-5D-Y utility scores at each follow-up time point compared with ABC-C scores, adjusting for disability level, accommodation and living situation and with site and patient identifier as random effects.

Sensitivity analyses are reported in the supplementary data 1.

The analysis was conducted in Stata version 14.

## Results

Out of the 246 participants recruited 108 were in one of the 11 intervention arm services and 137 were in one the 12 TAU arm services, with one participant excluded as not meeting the ABC-C inclusion threshold. The participants were working-age adults (median age 37 years, interquartile range (IQR) = 24–51), predominantly men (64%), with moderate or severe intellectual disability (83%) and a median total ABC-C score of 64 at baseline (IQR = 44–86).

Over two-thirds were receiving antipsychotic medication (67%) and had additional mental disorders (49% common mental disorders and 20% severe mental illness). A total of 50% of participants were on the autistic spectrum. Further details can be found in the main trial paper.^12,13^

At 36 months, complete cost and utility data were available for 78 (72%) participants in the intervention arm and for 102 (74%) in the control arm. The 12-month consort diagram is reported in supplementary Fig. 1.

### Cost of training in PBS and delivery

In total 26 therapists across the 11 services randomised to the intervention received training. The cost of the training was £397 per participant (see supplementary Table 2). Details of the activities carried out by therapists were available for 65 (60%) of the participants in the intervention arm for an average cost of £1201 (see supplementary Table 3) and a total average cost per participant of £1598 (training in and delivery of the PBS intervention).

### Resource use and costs

Descriptive statistics for resource use are reported in supplementary Tables 4 and 5. Total costs for each resource type are reported in Table 1 and are calculated using 35 imputed data-sets. The total cost of health and social care at 12 months (excluding the cost of training and delivering PBS) was £3603 (95% CI 2848–4358) in the intervention arm and £4051 (95% CI 3094–5008) in the TAU arm. The difference between the intervention and TAU arm was −£197 (95% CI −1140 to 697) adjusting for baseline costs, staffing ratio and clustering.

**Table 1:** Mean (s.e.) per participant resource use costs imputed and adjusting for baseline and covariates, not discounted, reported in 2014/2015 GBP

	Baseline, mean (s.e.)		Baseline to month 12, mean (s.e.)		Month 13 to between month 30 and month 36, mean (s.e.)		Total cost (12 months plus 36 months), mean (s.e.)		Adjusted 36-month difference	95% CI
	PBS	Control TAU	PBS	Control TAU	PBS	Control TAU	PBS	Control TAU		
Community costs including GPs, nursing, allied health and social care	1175 (119)	988 (210)	1467 (179)	1639 (158)	635 (87)	559 (99)	2034 (209)	2273 (180)	−317	−952 to 317
Mental health – secondary care	48 (9)	94 (35)	73 (14)	542 (346)	386 (379)	49 (36)	459 (382)	591 (349)	−31	−1097 to 1035
Physical health – secondary care	369 (83)	610 (111)	1327 (280)	985 (132)	613 (222)	382 (62)	1941 (405)	1367 (156)	825	0.34 to 1650
Medication costs	390 (105)	460 (92)	701 (130)	860 (237)	277 (74)	283 (53)	978 (157)	1143 (248)	−53	−475 to 369
Total health and social care	1982 (269)	2152 (218)	3603 (383)	4051 (486)	1924 (478)	1394 (143)	5526 (686)	5445 (524)	441	−1173 to 2056
Voluntary	43 (25)	16 (16)	21 (8)	10 (10)	0	0	21 (18)	10 (10)	14	−25 to 53
Private and out of pocket expenses	345 (96)	539 (140)	792 (163)	417 (100)	730 (157)	1606 (408)	1523 (252)	2023 (434)	−483	−1604 to 637
Accommodation	24 469 (1332)	19 217 (1309)	51 044 (2705)	39 552 (2710)	25 044 (1241)	19 940 (1320)	76 090 (3738)	59 490 (3954)	193	−2007 to 5872
Criminal justice	170 (54)	88 (37)	82 (37)	118 (55)	37 (17)	47 (19)	119 (44)	165 (70)	−84	−205 to 37
Unpaid/family carers	13 815 (3140)	22 821 (3225)	19 221 (4779)	35 158 (5210)	10 950 (2984)	24 526 (3554)	30 172 (7117)	59 683 (7982)	−15 430	−34 258 to 3400
Total societal costs	40 825 (2691)	45 143 (2687)	75 047 (3567)	78 937 (3816)	39 541 (2817)	48179 (2746)	114 588 (5241)	127 115 (5425)	−9694	−26 380 to 6992

PBS, positive behaviour support; TAU, treatment as usual; GP, general practitioner.

When the cost per participant including training in and delivery of PBS is added, the average difference in cost of the intervention compared with TAU at 12 months is £1401 (95% CI 458–2295). At 36 months the total mean health and social care costs per participant in the intervention arm were £5526 (95% CI 4173–6880; £5387 discounted, 95% CI 4091–6684) and £5445 in TAU (95% CI 4412–9478; £5348 discounted, 95% CI 4322–6372).

The adjusted difference in costs calculated from bootstrapping and multiple imputation for missing data at 36 months was £448 greater in the intervention than in the control arm (95% CI −£864 to £1821; £399 discounted, 95% CI −603 to 1724). At 36 months, the intervention total discounted cost is £1997 (95% CI 995–1770) when the cost of training and delivery of PBS is included.

At baseline, 52% of participants were receiving support from unpaid carers (family and significant others) who reported spending an average of 22 h (95% CI 12–32 h) caring for their relative in the intervention group and 36 h a week (95% CI 26–46 h) in TAU. At 12 months, informal care reduced to 15 h a week (95% CI 7–22 h) in the intervention group and to 27 h a week (95% CI 18–36 h) in TAU, an adjusted difference of −6 h a week (95% CI −4  to 15 h). However, by 36 months an increase in informal care was recorded of 16 h (95% CI 6–26 h) in the intervention arm and 33 h (95% CI 22–44 h) in TAU; an adjusted difference of −10 h (95% CI −23 to 3).

At 36 months, the intervention arm showed a reduction in societal costs of £14 229 (95% CI –26 774 to −1997) and when discounted −£13 633 (95% CI −25 755 to −1810). Descriptive statistics for resource use are reported in supplementary Tables 2 and 3.

### QALYs

The adjusted difference in QALYs between the two arms at 36 months was 0.184 in favour of the intervention (95% CI −0.080 to 0.449; 0.175 adjusted and discounted, 95% CI −0.068 to 0.418). At 12 months, the adjusted difference was 0.064 additional QALYs in the intervention arm (95% CI 0.004 to 0.124) (Table 2). Based on the multilevel model, there is a significant utility decrement of 0.002 (95% CI −0.003 to −0.001) for every 1-point change on the ABC-C.

**Table 2:** Utilities and quality-adjusted life-years (QALYs – unadjusted)

	Baseline		6 months		12 months		36 months		QALYs (36 months)	
	Intervention	Control	Intervention	Control	Intervention	Control	Intervention	Control	Intervention	Control
Mean	0.565	0.487	0.621	0.503	0.623	0.491	0.574	0.513	1.893	1.608
s.e.	0.037	0.033	0.034	0.032	0.032	0.029	0.033	0.032	0.093	0.083

### Cost-utility analysis

At 36 months the discounted incremental cost-effectiveness ratio from a health and social care cost perspective is £11 691 (£2046/0.175). At 12 months, the incremental cost per QALY gained of the intervention compared with TAU from a health and social care cost perspective is £21 538 (£1401/0.064) including the cost of training in and delivery of PBS. From a societal cost perspective, training in PBS and TAU dominates TAU only at both time points as it results in more QALYs for less cost.

**Fig. 1:**
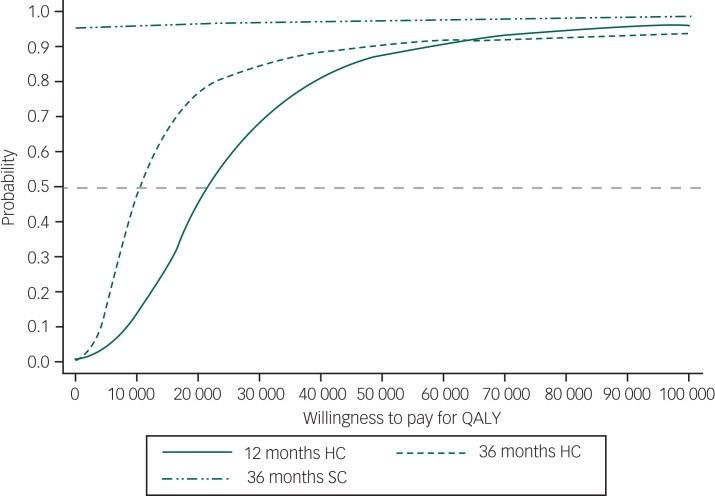
Cost-effectiveness acceptability curve for positive behaviour support training and delivery and treatment as usual (TAU) compared with TAU only over 36 months for a range of values of willingness to pay per quality-adjusted life-year (QALY) gained.

**Fig. 2:**
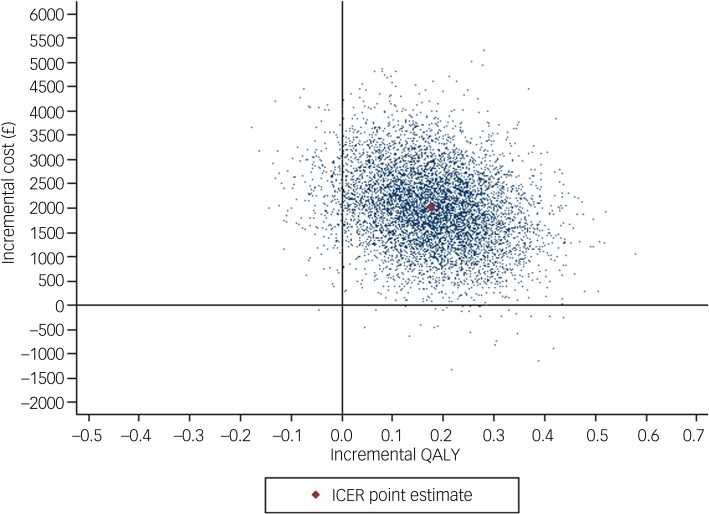
Cost-effectiveness plane of costs and quality-adjusted life-years (QALYs) for positive behaviour support training and delivery and treatment as usual (TAU) compared with TAU only from a healthcare cost perspective over 36 months.

At 36 months and from a health and social care cost perspective the intervention has an 85% probability of being cost-effective compared with TAU at a willingness to pay of £30 000 per QALY gained (Fig. 1). The cost-effectiveness plane is reported in Fig. 2. There is 97% probability that the intervention is cost-effective compared with current practice at willingness to pay (WTP) of £30 000 for a QALY gained from a societal perspective (see supplementary Fig. 2 for societal cost-effectiveness plane).

Results based on 7000 bootstraps of imputed data – discounted for costs occurring after 12 months. HC, healthcare; SC, wider societal costs.

ICER, incremental cost-effectiveness ratio.

The conclusions of the analysis remain the same in the sensitivity analyses (see supplementary data 1).

## Discussion

To our knowledge, this is the first health economic evaluation of staff training in PBS and the delivery of PBS in routine care as part of a multicentre, pragmatic RCT. At 36 months the intervention was cost-effective from both a healthcare and wider societal cost perspective, with an 85% and 97% probability, respectively of being cost-effective at a WTP of £30 000 for a QALY gained. The 36-month duration was chosen to capture costs and QALYs for the longest time horizon possible rather than the 12-month primary end-point for the clinical effectiveness trial. Ideally, we would use a decision model to project costs and QALYs over the lifetime of the participants, but limited evidence in the wider published literature for the long-term impact of behavioural interventions for adults with intellectual disability and behaviours that challenge precludes such approaches.

Although the cost of training in and delivery of PBS is relatively high for a psychological intervention at £1598 per participant, this is compensated for by a greater number of QALYs in the intervention arm compared with TAU. Intensive clinical interventions commonly cost more than routine practice and as a result public bodies responsible for allocating healthcare resources need a way of deciding if the additional benefits are worth the costs. In the English NHS this is guided by NICE. To calculate benefits, NICE recommends the use of the EQ-5D to calculate QALYs so that decisions can be standardised across different clinical areas and to use a decision threshold for adopting treatments of £30 000 for a QALY gained.^14^ The QALY gain of 0.175 (discounted) at 36 months and the significant increase in QALYs at 12 months is a key driver in the findings from the cost-utility analysis, particularly given that the intervention cost significantly more. Nevertheless, this finding should be considered with caution as QALYs are a secondary trial outcome. There is limited methodological work on the suitability of the EQ-5D in intellectual disability and with proxies. We found that there was a significant relationship between the primary outcome of ABC-C and the proxy completed EQ-5D-Y, suggesting that it is responsive to changes in challenging behaviour.

From a societal cost perspective, the cost of the intervention was compensated by a reduction in the time informal carers (family and close others) spent caring for participants. Although encouraging, this was assessed by a single question asking the number of hours of informal care in a typical week in the past 6 months.

### Comparison with other studies

Staff training in PBS in community intellectual disability services forms part of the increased emphasis on person-centred care for people with intellectual disability in the UK and other high-income countries.^10,29^ The evidence for the cost-effectiveness of PBS and other interventions for behaviours that challenge in adults with intellectual disability are limited, particularly economic evaluations that include both costs and QALY.^9,30^ One pilot RCT of a specialist service delivering applied behavioural analysis (ABA) in one area in England^31^ found a significant difference in ABC-C total and domain scores in the intervention arm at 6 months. Service use showed that ABA on average reduced the costs by £2900 per participant compared with TAU (95% CI −£6788 to 987). The study demonstrated clinical effectiveness as well as potential cost savings, but did not include a health-related quality-of-life measure and hence QALYs could not be evaluated.^31^

A few non-RCT studies have included economic evaluations. Hudson *et al*^32^ carried out a cost–benefit analysis of a specialist community team indicating that the cost of an intervention was Australian $5725; Grey and McClean^33^ estimated that training in PBS saved 2000 euros per participant.

Felce and colleagues^34^ reported the resource use of a group intervention (cognitive–behavioural therapy for anger management). This was a multicentre RCT and thus more comparable with our study. The intervention was manualised but carried out by support staff who were supervised for the trial duration. The intervention was found to be potentially cost saving but there was considerable cost heterogeneity observed associated with residential accommodation. However, the study did not include health-related quality-of-life measures, therefore, QALYs could not be calculated.

Iemmi *et al*^35^ carried out a Delphi exercise to calculate the cost of PBS, which they estimated to be £2564 per week (updated to 2016/2017 costs^36^). This is significantly greater than the cost of PBS training and delivery found in our study. The vignettes used by Iemmi *et al*^35^ indicated that all participants lived in residential care whereas in our study 43% of participants lived in residential care.

### Strengths and limitations

A key strength of this study is that it includes a full health economic evaluation compliant with NICE guidance. The long follow-up duration (36 months) was also a strength, with other studies of psychosocial interventions in the field of intellectual disability rarely following participants up long enough to capture the potential long-term impact.^37^

Although the long duration of follow-up is a strength, one of the limitations was that the services in the TAU arm were able to access PBS training after having completed the main study at 12 months. This coincided with the implementation of a large-scale training programme in PBS on the recommendation of NHS England as part of the transforming care programme.^38^ By 36 months all services except two in the control arm had implemented some form of specialist behavioural support. We have maintained the intention-to-treat analysis as prespecified in the analysis plan, with any differences between groups most likely because of randomisation to PBS staff training provided at the start of the study.

Furthermore, the 36-month CSRI follow-up assessment only enquired about service use in the past 6 months, as recall durations longer than 6 months are unreliable.^39^ As a result, we were unable to capture resource use between 12 and 30 months. The EQ-5D was also only collected at 36 months, potentially missing any changes that may have occurred between 12 months and 36 months.

Sixty-six participants had data missing for at least one time point for either resource use or EQ-5D. Although we have attempted to address this by multiple imputation, the approach will not have completely overcome the potential bias implicit in incomplete follow-up data.

Calculating the cost of the training in PBS and of its delivery also presents some limitations. It is likely that our estimate of the costs of staff training is conservative given that the cost per participant is calculated as the total cost of training divided by the number of participants enrolled in the study. It is possible that more people with intellectual disability than those enrolled in the study may have benefited. The costs of training may also be lower if some items were removed or reduced, for example, mentoring or travel costs. However, our training costs may underestimate some additional costs as they do not account for the cost of training additional staff as a result of therapist turnover. Overall our training cost of £2142 per staff member trained (see supplementary Table 2: £42,842 divided by 20 participants) is at the upper end of the cost for part-time postgraduate courses in PBS that range from £800 to £3100 per person trained^40^ and includes the fees of delivering the course only and does not include costs associated with time off work for clinical staff to attend the course. The estimate used in this study also includes travel and expenses, which are not included in the other course estimates.

Not all staff completed documentation on the amount of time they spent delivering PBS-related activities. There was also less-than-optimal delivery of PBS, with 30% of participants receiving all elements of the PBS approach.^12^ This is likely to reflect realistic implementation of PBS in services, and hence the results reported are more likely to equate with those seen if PBS plus staff training is implemented in line with the methods used in this trial.

Although costing for accommodation type was based on broad assumptions including the staff/patient ratio for difference accommodation types and the number of rooms, there was little evidence of changes in participants' accommodation throughout the study; only 13 participants (5%) moved accommodation because of poor care in their previous home or because of changing health needs. We were unable to provide any estimates of the impact on employment or receipt of social security as a result of the intervention, given that employment of people with intellectual disability is about 5% nationally and other research suggests that care costs are fairly static.^17^

### Implications

In conclusion, this study adds to the evidence base for the cost-effectiveness of health staff training in PBS to treat adults with intellectual disability who display behaviour that challenges. Despite the lack of clinical effectiveness,^12,13^ there was a positive impact on health-related quality of life and less burden on informal care as shown by the sustained reduction in hours of care over time in the intervention arm. Decisions about health resource allocation should be based on the relative benefits and costs of interventions although these cannot be the sole criteria used. In light of the study finding that staff training in PBS did not reduce challenging behaviour above TAU,^12,13^ it is essential that services, trainers and policymakers reach consensus as to whether PBS ought to be delivered by specialists or whether other ‘light touch’ approaches may be acceptable. Despite the 85% probability of being cost-effective at £30 000 WTP per QALY gained, training in PBS is unlikely to tackle serious organisational barriers that practitioners need to overcome if PBS skills are to be properly implemented.

Future studies testing complex interventions should place greater focus on identifying active ingredients of interventions likely to add therapeutic and cost–benefit and consider measuring additional outcomes that may be more relevant to adults with intellectual disability and their families.

## Data Availability

Authors have access to the original study data. Data is available on request.

## Supplementary material

For supplementary material accompanying this paper visit http://dx.doi.org/10.1192/bjo.2020.1. click here to view supplementary material
