# Supplementary material for: Staff training in positive behaviour support for behaviour that challenges in people with intellectual disability: cost-utility analysis of a cluster randomised controlled trial
Source: BJPsych Open. 2020 Feb 5;6(2):e15. doi: 10.1192/bjo.2020.1 (PMC7176891; doi:10.1192/bjo.2020.1)
Supplement: Supplementary file 1 [file S2056472420000010sup001.docx]

**Supplementary material**

Supplementary Table 1: Unit costs for health, social care and criminal justice. All costs are reported in GBP 2014/2015.^1^

|  | **Unit Cost (Per contact)** | **Source** |
| --- | --- | --- |
| **Community care** |  |  |
| GP (surgery) | 33 | PSSRU |
| GP (Home) | 68 | PSSRU |
| GP (Phone) | 20 | PSSRU |
| District Nurse | 39 | PSSRU, Ball et al 2014 |
| Mental health Nurse | 17.5 | PSSRU, Ball et al 2014 |
| Learning disabilities nurse | 17.5 | PSSRU |
| Psychiatrist | 39 | PSSRU, Cruz et al 2013 |
| Psychologist | 52 | PSSRU, Pomerantz et al 2009 |
| Social Worker | 55 | PSSRU |
| Occupational therapist | 33 | PSSRU, Renforth et al 2014 |
| Art/Drama/Music therapy | 68 | Reference costs |
| Alternative therapy | 85 | Reference costs |
| Counsellor | 44 | PSSRU |
| Physiotherapist | 52 | Reference costs |
| Dentist | 85 | Reference costs |
| Speech and language therapist | 84 | Reference costs |
| Community support worker | 51 | PSSRU |
| Chiropodist/podiatrist | 44 | Reference costs |
| Optician | 97 | Reference costs |
| Behavioural therapist | 85 | Reference costs |
| **Acute and specialist care** |  |  |
| Mental Health (per bed day) | 223 | PSSRU |
| Short non-elective stay | 608 | PSSRU |
| Long non-elective stay | 2863 | PSSRU |
| Planned (elective) acute care | 3405 | PSSRU |
| Critical care stay | 1207 | Reference costs |
| Mental Health (outpatient) | 107 | PSSRU |
| General medical outpatient | 112 | PSSRU |
| Day Case | 704 | PSSRU |
| A&E attendance (admitted) | 167 | Reference costs |
| A&E attendance (not admitted) | 108 | Reference costs |
| **Criminal Justice** |  |  |
| Police contact (arrested) | 267 | Heslin et al 2017 |
| Police contact (not-arrested) | 841 | Heslin et al 2017 |
| Learning disabilities assessment | 284 | PSSRU |
| Probation | 2690 | Hayhurst et al 2015 |

Supplementary Fig. 1. Trial Consort Diagram (12 months).^1-2^

**Number of teams recruited**

N_t_ = 28

**Teams Excluded**

Refuse to take part N_t_ = 5

**Allocated to Intervention**

N_t_=11

N_su_=108

N_pc_= 89; N_fc_=19

N_pc=_ ; N_fc=_

Deliver PBS training

Lost to follow up; refused N_su_=3

Follow up too early/late N_su_=17

Lost to follow up; refused N_su_=3

Follow up too early/late N_su_=7

Lost to follow-up; refused N_su_=1

Follow up too early/late N_su_=3

**Allocated to TAU**

N_t_=12

N_su_=137

N_pc_= 86; N_fc_=50

**Number of teams**, N_t_=23

**Service users screened**, N_su_= 382

**Service users recruited,**

N_su_=246

**Service User Excluded,** N_su_= 136

Reached recruitment target: 52,

No consent provided: 42,

Ineligible: 22,

Moved out of area: 7,

Adverse event: 5,

Uncontactable: 4,

Not screened in time: 4

**Service User Excluded**

Ineligible N_su_=1

Lost to follow-up; refused N_su_=2

Follow up too early/late N_su_=5

**Time 2 (6 months) assessments**

N_su_=117

N_pc_= 76; N_fc_=39

**Time 2 (6 months) assessments**

N_su=_98

N_pc=_ 81; N_fc_=17

**Time 3 (12 months) assessments**

N_su_=100

N_pc_=84 ;N_fc_=16

**Time 3 (12 months) assessments**

N_su_= 125

N_pc=_ 82; N_fc_=42

**Key**

N_t_ Number of teams N_su_Number of service users

N_pc_ Number of paid carers N_fc_ Number of family carers

##### Supplementary Table 2. Cost of training activities in the trial^1^

| **Activity** | **Total cost** |
| --- | --- |
| 1. Preparation and printing of Training Manual & Material | £873 |
| 2. Workshops |  |
| I. 2 days, 4 trainers | £6,669 |
| II. 3 days, 3 trainers |  |
| III. 2 days, 3 trainers |  |
| 3. Preparation for workshops |  |
| (6 days x 7 hours x £50) | £2,100 |
| 4. Reading and commenting on participant assessments and PBS plans between workshops I & ii & ii & iii |  |
| (4 hours x 2 workshops x 20 participants x £50) | £8,000 |
| 5. Mentoring Post-Workshops |  |
| 2 hours per month x 12 months x  20 participants x £50 | £24,000 |
| 6. Travel & Expenses | £1,200 |
| **Grand Total** | **£42,842** |
| **Cost per patient (assuming 108 patients in intervention arm)** | **£397** |

##### Supplementary Table 3. Cost of delivering PBS^1^

| **Activity** | **Average Hours** | **Cost of Band 6 NHS staff** |
| --- | --- | --- |
| Assessment - direct observation of client | 3.6 | £157 |
| Assessment - contact with staff team, parents/carers, wider system | 5.2 | £227 |
| Assessment - indirect work | 3.8 | £169 |
| Intervention - direct contact with client | 2.4 | £104 |
| Intervention - contact with staff team, parents/carers, wider system | 3.2 | £143 |
| Report writing and other admin not accounted for elsewhere | 6.9 | £302 |
| Other work not accounted for in other columns | 2.7 | £118 |
| **Total hours spent on case** | **27.3** | **£1,201** |

**Supplementary** **Table 4. Resource use-Baseline to 12 months.^1^**

|  |  | **Baseline** |  | **6 months** |  | **12 months** |  |
| --- | --- | --- | --- | --- | --- | --- | --- |
|  |  | PBS | Control | PBS | Control | PBS | Control |
|  |  | 108 | 136 | 103 | 130 | 103 | 132 |
|  |  |  |  |  |  |  |  |
| GP (surgery) | Yes (n) | 88 | 103 | 85 | 105 | 83 | 103 |
|  | Yes (%) | 82% | 76% | 83% | 81% | 81% | 78% |
|  | For those that used the service |  |  |  |  |  |  |
|  | Mean | 5.1 | 4 | 4.3 | 4.2 | 5 | 3.5 |
|  | SD | 6.6 | 4 | 8.4 | 6.6 | 11 | 3.3 |
|  |  |  |  |  |  |  |  |
| GP (Home) | Yes (n) | 14 | 30 | 12 | 23 | 13 | 21 |
|  | Yes (%) | 13% | 22% | 12% | 18% | 13% | 16% |
|  | For those that used the service |  |  |  |  |  |  |
|  | Mean | 2 | 3.3 | 2.6 | 3.4 | 1.5 | 2.1 |
|  | SD | 1.7 | 4.8 | 2.8 | 5.6 | 0.9 | 2.5 |
|  |  |  |  |  |  |  |  |
| GP (Phone) | Yes (n) | 29 | 50 | 24 | 46 | 20 | 28 |
|  | Yes (%) | 27% | 37% | 24% | 36% | 20% | 29% |
|  | For those that used the service |  |  |  |  |  |  |
|  | Mean | 2.4 | 4.1 | 3.6 | 6.4 | 2.6 | 2.7 |
|  | SD | 2.1 | 7.4 | 4.8 | 15.4 | 1.7 | 2.5 |
|  |  |  |  |  |  |  |  |
| Community/District Nurse | Yes (n) | 24 | 46 | 23 | 31 | 33 | 31 |
|  | Yes (%) | 22% | 34% | 23% | 24% | 32% | 23% |
|  | For those that used the service |  |  |  |  |  |  |
|  | Mean | 4 | 11.6 | 5.7 | 10.2 | 6 | 5 |
|  | SD | 5.5 | 33 | 6.3 | 34.1 | 13.1 | 8.6 |
|  |  |  |  |  |  |  |  |
| Community Psychiatric nurse | Yes (n) | 26 | 12 | 17 | 14 | 17 | 15 |
|  | Yes (%) | 24% | 9% | 17% | 11% | 17% | 11% |
|  | For those that used the service |  |  |  |  |  |  |
|  | Mean | 5.2 | 3.1 | 5.4 | 2.9 | 3.5 | 4.3 |
|  | SD | 7.6 | 3.1 | 6.1 | 3.2 | 4.1 | 5.6 |
|  |  |  |  |  |  |  |  |
| Learning disabilities nurse | Yes (n) | 38 | 40 | 26 | 22 | 24 | 27 |
|  | Yes (%) | 35% | 29% | 26% | 17% | 23% | 20% |
|  | For those that used the service |  |  |  |  |  |  |
|  | Mean | 4.3 | 12.5 | 4.4 | 20.2 | 4.4 | 2.9 |
|  | SD | 4.7 | 35.9 | 5.9 | 54.2 | 4.7 | 4.7 |
|  |  |  |  |  |  |  |  |
| Psychiatrist | Yes (n) | 79 | 88 | 66 | 81 | 60 | 78 |
|  | Yes (%) | 73% | 65% | 65% | 63% | 58% | 59% |
|  | For those that used the service |  |  |  |  |  |  |
|  | Mean | 2 | 1.8 | 1.7 | 2 | 1.4 | 2.1 |
|  | SD | 2.9 | 1.5 | 1.3 | 1.8 | 0.9 | 1.8 |
|  |  |  |  |  |  |  |  |
| Psychologist | Yes (n) | 16 | 26 | 8 | 23 | 13 | 29 |
|  | Yes (%) | 15% | 19% | 8% | 18% | 13% | 22% |
|  | For those that used the service |  |  |  |  |  |  |
|  | Mean | 3.7 | 4.1 | 4.4 | 2.3 | 2.2 | 3.1 |
|  | SD | 6.3 | 6.4 | 5.6 | 2.4 | 2.2 | 4.6 |
|  |  |  |  |  |  |  |  |
| Social Worker/Care manager | Yes (n) | 64 | 57 | 48 | 63 | 55 | 60 |
|  | Yes (%) | 59% | 42% | 47% | 48% | 53% | 46% |
|  | For those that used the service |  |  |  |  |  |  |
|  | Mean | 5.2 | 2.5 | 2 | 2.3 | 2.6 | 2.4 |
|  | SD | 23.2 | 2.5 | 2 | 3.5 | 7.7 | 2.5 |
|  |  |  |  |  |  |  |  |
| Occupational Therapist | Yes (n) | 20 | 17 | 17 | 22 | 12 | 22 |
|  | Yes (%) | 19% | 13% | 17% | 17% | 12% | 17% |
|  | For those that used the service |  |  |  |  |  |  |
|  | Mean | 2.2 | 3.2 | 3.9 | 2.5 | 2.5 | 1.9 |
|  | SD | 2.3 | 2.7 | 6 | 1.9 | 2.2 | 1.5 |
|  |  |  |  |  |  |  |  |
| Art/drama/music therapy | Yes (n) | 7 | 5 | 9 | 6 | 8 | 7 |
|  | Yes (%) | 6% | 4% | 9% | 5% | 8% | 5% |
|  | For those that used the service |  |  |  |  |  |  |
|  | Mean | 20.6 | 16.2 | 18.7 | 21.9 | 12.25 | 11.1 |
|  | SD | 17.1 | 11.8 | 9.7 | 17.2 | 18.7 | 6.6 |
|  |  |  |  |  |  |  |  |
| Alternative Therapist | Yes (n) | 6 | 5 | 1 | 8 | 1 | 2 |
|  | Yes (%) | 6% | 4% | 1% | 6% | 1% | 2% |
|  | For those that used the service |  |  |  |  |  |  |
|  | Mean | 14.8 | 18.8 | 13 | 16 | 48 | 19 |
|  | SD | 12.1 | 10 |  | 9.3 |  | 9.9 |
|  |  |  |  |  |  |  |  |
| Counsellor | Yes (n) | 2 | 2 | 0 | 2 | 0 | 0 |
|  | Yes (%) | 2% | 1% | 0% | 2% | 0% | 0% |
|  | For those that used the service |  |  |  |  |  |  |
|  | Mean | 4.7 | 6 |  | 13.5 |  |  |
|  | SD | 6.4 |  |  | 17.7 |  |  |
|  |  |  |  |  |  |  |  |
| Physiotherapy | Yes (n) | 6 | 21 | 6 | 16 | 7 | 8 |
|  | Yes (%) | 6% | 15% | 6% | 12% | 7% | 6% |
|  | For those that used the service |  |  |  |  |  |  |
|  | Mean | 2.7 | 4.8 | 7.7 | 6.1 | 6.3 | 3 |
|  | SD | 7.1 | 8.8 | 9.6 | 8.4 | 7.9 | 3.8 |
|  |  |  |  |  |  |  |  |
| Dentist | Yes (n) | 81 | 77 | 72 | 81 | 69 | 79 |
|  | Yes (%) | 75% | 57% | 71% | 63% | 67% | 60% |
|  | For those that used the service |  |  |  |  |  |  |
|  | Mean | 1.7 | 1.5 | 1.5 | 1.7 | 1.5 | 1.5 |
|  | SD | 1.5 | 0.9 | 1.5 | 1.8 | 1 | 1 |
|  |  |  |  |  |  |  |  |
| Speech and language therapy | Yes (n) | 14 | 14 | 14 | 26 | 17 | 26 |
|  | Yes (%) | 13% | 10% | 14% | 20% | 17% | 20% |
|  | For those that used the service |  |  |  |  |  |  |
|  | Mean | 2.2 | 2.1 | 5.6 | 6 | 3.5 | 2.1 |
|  | SD | 2 | 2 | 6.7 | 7.9 | 2.9 | 1.3 |
|  |  |  |  |  |  |  |  |
| Community support worker | Yes (n) | 6 | 9 | 6 | 9 | 4 | 15 |
|  | Yes (%) | 6% | 7% | 6% | 7% | 4% | 12% |
|  | For those that used the service |  |  |  |  |  |  |
|  | Mean | 89.7 | 59.4 | 99.7 | 54.7 | 98.3 | 79.7 |
|  | SD | 79.7 | 122 | 76.7 | 58.7 | 97.8 | 106.2 |
|  |  |  |  |  |  |  |  |
| Acute care and specialist services |  |  |  |  |  |  |  |
|  |  |  |  |  |  |  |  |
| Mental Health Inpatient | Yes (n) | 0 | 2 | 0 | 1 | 0 | 2 |
|  | Yes (%) | 0% | 1% | 0% | 1% | 0% | 2% |
|  | For those with an admission |  |  |  |  |  |  |
| Number of admissions | Mean |  | 1 |  | 1 |  | 1 |
|  | SD |  | 0 |  |  |  |  |
| Number of bed days | Mean |  | 12 |  | 30 |  | 110 |
|  | SD |  | 12.7 |  |  |  | 110.3 |
|  |  |  |  |  |  |  |  |
| General medical planned admission | Yes (n) | 0 | 3 | 1 | 0 | 2 | 1 |
|  | Yes (%) | 0% | 2% | 1% | 0% | 2% | 1% |
|  | For those with an admission |  |  |  |  |  |  |
| Number of admissions | Mean |  | 1 | 2 |  | 1 | 1 |
|  | SD |  | 0 |  |  | 1 |  |
| Number of bed days | Mean |  | 1 | 8 |  | 7.7 | 0 |
|  | SD |  | 0 |  |  | 11.6 |  |
|  |  |  |  |  |  |  |  |
| General medical unplanned admission | Yes (n) | 5 | 11 | 4 | 5 | 4 | 5 |
|  | Yes (%) | 5% | 8% | 4% | 4% | 4% | 4% |
|  | For those with an admission |  |  |  |  |  |  |
| Number of admissions | Mean | 2.5 | 1.3 | 2.25 | 1.9 | 1.25 | 1 |
|  | SD | 1.9 | 0.7 | 1.5 | 0.6 | 0.5 | 0 |
| Number of bed days | Mean | 5.3 | 4.2 | 6.25 | 6.4 | 6 | 4 |
|  | SD | 4.8 | 5.8 | 3.9 | 6.2 | 7.4 | 5.6 |
|  |  |  |  |  |  |  |  |
| Medical ICU/HDU | Yes (n) | 2 | 1 | 0 | 1 | 0 | 0 |
|  | Yes (%) | 2% | 1% | 0% | 1% | 0% | 0% |
|  | For those with an admission |  |  |  |  |  |  |
| Number of admissions | Mean | 1 | 2 |  | 1 |  |  |
|  | SD | 0 |  |  |  |  |  |
| Number of bed days | Mean | 4.5 | 4 |  | 17 |  |  |
|  | SD | 2.1 |  |  |  |  |  |
|  |  |  |  |  |  |  |  |
|  |  |  |  |  |  |  |  |
| Physical health related A&E attendance | Yes (n) | 20 | 31 | 12 | 32 | 15 | 18 |
|  | Yes (%) | 19% | 23% | 12% | 25% | 15% | 14% |
|  | For those that used the service |  |  |  |  |  |  |
|  | Mean | 1.6 | 1.8 | 2.2 | 1.6 | 3.4 | 1.4 |
|  | SD | 1 | 1.5 | 22 | 1.7 | 7.5 | 1 |
|  |  |  |  |  |  |  |  |
| Mental health related A&E attendance | Yes (n) | 3 | 1 | 1 | 3 | 2 | 2 |
|  | Yes (%) | 3% | 1% | 1% | 2% | 2% | 2% |
|  | For those that used the service |  |  |  |  |  |  |
|  | Mean | 7 | . | 26 | 7.5 | 1 | 1.5 |
|  | SD | 1.4 | . |  | 9.2 | 0 | 0.7 |
|  |  |  |  |  |  |  |  |
| Psychiatric outpatient appointment | Yes (n) | 28 | 43 | 31 | 49 | 21 | 40 |
|  | Yes (%) | 26% | 32% | 31% | 38% | 21% | 30% |
|  | For those that used the service |  |  |  |  |  |  |
|  | Mean | 1.7 | 1.7 | 1.4 | 1.6 | 1.6 | 1.4 |
|  | SD | 1 | 1 | 1.1 | 1.3 | 1.2 | 0.7 |
|  |  |  |  |  |  |  |  |
| Day patient procedure | Yes (n) | 13 | 40 | 24 | 32 | 32 | 39 |
|  | Yes (%) | 12% | 29% | 24% | 25% | 31% | 30% |
|  | For those that used the service |  |  |  |  |  |  |
|  | Mean | 2.1 | 1.6 | 1.9 | 1.5 | 2.2 | 1.8 |
|  | SD | 1.3 | 0.8 | 1.6 | 1 | 2.2 | 1.3 |
|  |  |  |  |  |  |  |  |
| Medical outpatient appointment | Yes (n) | 25 | 36 | 21 | 27 | 24 | 24 |
|  | Yes (%) | 23% | 27% | 21% | 21% | 23% | 18% |
|  | For those that used the service |  |  |  |  |  |  |
|  | Mean | 2.4 | 2.2 | 3 | 2.2 | 2.6 | 3.3 |
|  | SD | 2.7 | 3 | 2.4 | 3.7 | 2 | 5 |

**Supplementary Table 5**: Resource use: 30 months to 36 months

|  |  | **36 months** |  |
| --- | --- | --- | --- |
|  |  | PBS | Control |
|  |  | 79 | 105 |
|  |  |  |  |
| **GP (surgery)** | Yes (n) | 63 | 78 |
|  | Yes (%) | 80% | 74% |
|  | For those that used the service |  |  |
|  | Mean | 3.9 | 3.1 |
|  | SD | 6.6 | 3.9 |
|  |  |  |  |
| **GP (Home)** | Yes (n) | 7 | 13 |
|  | Yes (%) | 9% | 12% |
|  | For those that used the service |  |  |
|  | Mean | 1.4 | 2.3 |
|  | SD | 0.5 | 2.2 |
|  |  |  |  |
| **GP (Phone)** | Yes (n) | 11 | 16 |
|  | Yes (%) | 14% | 15% |
|  | For those that used the service |  |  |
|  | Mean | 1.9 | 5.9 |
|  | SD | 0.9 | 7.7 |
|  |  |  |  |
| **Community/District Nurse** | Yes (n) | 18 | 29 |
|  | Yes (%) | 23% | 28% |
|  | For those that used the service |  |  |
|  | Mean | 15.4 | 7.9 |
|  | SD | 41.9 | 33.5 |
|  |  |  |  |
| **Community Psychiatric nurse** | Yes (n) | 2 | 4 |
|  | Yes (%) | 3% | 4% |
|  | For those that used the service |  |  |
|  | Mean | 6 | 4.5 |
|  | SD | 1.4 | 3.5 |
|  |  |  |  |
| **Learning disabilities nurse** | Yes (n) | 10 | 18 |
|  | Yes (%) | 12% | 17% |
|  | For those that used the service |  |  |
|  | Mean | 1.9 | 7.7 |
|  | SD | 1.5 | 12.9 |
|  |  |  |  |
| **Psychiatrist** | Yes (n) | 35 | 62 |
|  | Yes (%) | 44% | 59% |
|  | For those that used the service |  |  |
|  | Mean | 1.6 | 1.9 |
|  | SD | 1.2 | 1.8 |
|  |  |  |  |
| **Psychologist** | Yes (n) | 11 | 12 |
|  | Yes (%) | 14% | 11% |
|  | For those that used the service |  |  |
|  | Mean | 2.2 | 2.7 |
|  | SD | 1.7 | 1.9 |
|  |  |  |  |
| **Social Worker/Care manager** | Yes (n) | 29 | 45 |
|  | Yes (%) | 37% | 43% |
|  | For those that used the service |  |  |
|  | Mean | 1.5 | 3 |
|  | SD | 1.2 | 5.4 |
|  |  |  |  |
| **Occupational Therapist** | Yes (n) | 11 | 14 |
|  | Yes (%) | 14% | 13% |
|  | For those that used the service |  |  |
|  | Mean | 4.3 | 2.9 |
|  | SD | 7.9 | 2.1 |
|  |  |  |  |
| **Art/drama/music therapy** | Yes (n) | 11 | 17 |
|  | Yes (%) | 14% | 16% |
|  | For those that used the service |  |  |
|  | Mean | 12.3 | 16.2 |
|  | SD | 11.4 | 9.5 |
|  |  |  |  |
| **Alternative Therapist** | Yes (n) | 8 | 8 |
|  | Yes (%) | 10% | 8% |
|  | For those that used the service |  |  |
|  | Mean | 17.8 | 20.1 |
|  | SD | 9 | 15.9 |
|  |  |  |  |
| **Counsellor** | Yes (n) | 0 | 0 |
|  | Yes (%) | 0% | 0% |
|  | For those that used the service |  |  |
|  | Mean |  |  |
|  | SD |  |  |
|  |  |  |  |
| **Physiotherapy** | Yes (n) | 5 | 12 |
|  | Yes (%) | 6% | 11% |
|  | For those that used the service |  |  |
|  | Mean | 3.8 | 2.2 |
|  | SD | 5.2 | 1.4 |
|  |  |  |  |
| **Dentist** | Yes (n) | 48 | 63 |
|  | Yes (%) | 60% | 60% |
|  | For those that used the service |  |  |
|  | Mean | 1.2 | 1.2 |
|  | SD | 0.6 | 0.6 |
|  |  |  |  |
| **Speech and language therapy** | Yes (n) | 3 | 16 |
|  | Yes (%) | 4% | 15% |
|  | For those that used the service |  |  |
|  | Mean | 4.7 | 3.7 |
|  | SD | 2.3 | 6.4 |
|  |  |  |  |
| **Community support worker** | Yes (n) | 8 | 10 |
|  | Yes (%) | 10% | 10% |
|  | For those that used the service |  |  |
|  | Mean | 65.3 | 176.9 |
|  | SD | 49.7 | 156 |
|  |  |  |  |
| **Acute care and specialist services** |  |  |  |
|  |  |  |  |
| **Mental Health Inpatient** | Yes (n) | 1 | 0 |
|  | Yes (%) | 1% | 0% |
|  | For those with an admission |  |  |
| **Number of admissions** | Mean | 1 |  |
|  | SD |  |  |
| **Number of bed days** | Mean | 120 |  |
|  | SD |  |  |
|  |  |  |  |
| **Mental Health Rehabilitation** | Yes (n) | 0 | 1 |
|  | Yes (%) | 0% | 1% |
|  | For those with an admission |  |  |
| **Number of admissions** | Mean |  | 1 |
|  | SD |  |  |
| **Number of bed days** | Mean |  | 42 |
|  | SD |  |  |
|  |  |  |  |
| **General medical planned admission** | Yes (n) | 0 | 0 |
|  | Yes (%) | 0% | 0% |
|  | For those with an admission |  |  |
| **Number of admissions** | Mean |  |  |
|  | SD |  |  |
| **Number of bed days** | Mean |  |  |
|  | SD |  |  |
|  |  |  |  |
| **General medical unplanned admission** | Yes (n) | 4 | 9 |
|  | Yes (%) | 5% | 9% |
|  | For those with an admission |  |  |
| **Number of admissions** | Mean | 1.25 | 1 |
|  | SD | 0.5 | 0 |
| **Number of bed days** | Mean | 20.8 | 6.1 |
|  | SD | 33 | 5.1 |
|  |  |  |  |
| **Medical ICU/HDU** | Yes (n) | 0 | 0 |
|  | Yes (%) | 0% | 0% |
|  | For those with an admission |  |  |
| **Number of admissions** | Mean |  |  |
|  | SD |  |  |
| **Number of bed days** | Mean |  |  |
|  | SD |  |  |
|  |  |  |  |
|  |  |  |  |
| **Physical health related A&E attendance** | Yes (n) | 9 | 22 |
|  | Yes (%) | 11% | 21% |
|  | For those that used the service |  |  |
|  | Mean | 1.3 | 1.6 |
|  | SD | 0.7 | 2 |
|  |  |  |  |
| **Mental health related A&E attendance** | Yes (n) | 2 | 1 |
|  | Yes (%) | 3% | 1% |
|  | For those that used the service |  |  |
|  | Mean | 75.5 | 1 |
|  | SD | 105.4 |  |
|  |  |  |  |
| **Psychiatric outpatient appointment** | Yes (n) | 3 | 8 |
|  | Yes (%) | 4% | 7% |
|  | For those that used the service |  |  |
|  | Mean | 1 | 1.25 |
|  | SD | 0 | 0.5 |
|  |  |  |  |
| **Day patient procedure** | Yes (n) | 16 | 27 |
|  | Yes (%) | 20% | 26% |
|  | For those that used the service |  |  |
|  | Mean | 1.8 | 1.3 |
|  | SD | 1.6 | 0.8 |
|  |  |  |  |
| **Medical outpatient appointment** | Yes (n) | 13 | 16 |
|  | Yes (%) | 16% | 15% |
|  | For those that used the service |  |  |
|  | Mean | 1.5 | 1.4 |
|  | SD | 0.9 | 0.8 |

**Supplementary** **Figure 2: Cost-effectiveness plane of costs and QALYs for PBS training and delivery compared to TAU from a societal cost perspective over 36 months.**

**
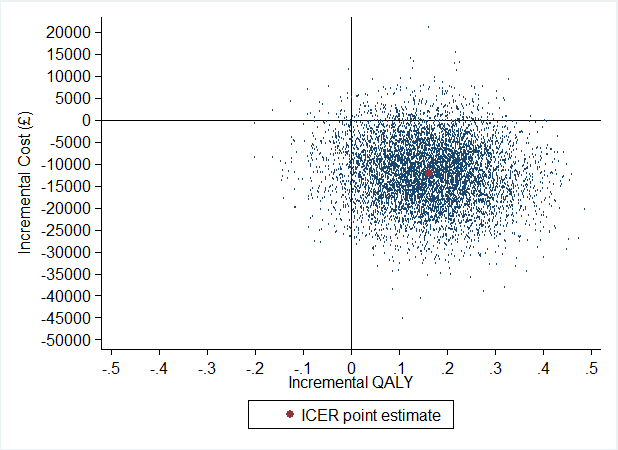
**

**Supplement Data 1**

**Sensitivity analyses:**

The EQ-5D-Y was self-completed by 11 participants (7 in the intervention and 4 in the TAU arm) at baseline, 6 months, 12 months and 36 months, therefore we were able to generate QALYs; The adjusted average QALYs over 36 months, was 2.45 (95% CI 2.39 to 2.51) for the intervention and 1.93 (95% CI 1.82 to 2.05) for the TAU arms with a mean, baseline adjusted difference of 0.516 QALYs (95% CI 0.460 to 0.573). The adjusted average QALYs over 12 months for 26 participants (14 in the intervention and 12 in the TAU arms respectively), was 0.782 (95% CI 0.749 to 0.815) for the intervention and 0.735 (95% CI 0.639 to 0.830) for the TAU arms with a mean, baseline adjusted difference of 0.043 QALYs (95% CI -0.075 to 0.170). This equates to a mean incremental cost per QALY gained of £3,965 from the health care perspective at 36 months and £32,581 from a societal cost perspective.

We have also reported the total cost of training in PBS for therapists at different levels of seniority assuming a range of caseloads to estimate the cost per participant of training i.e. if a staff member has a higher caseload the cost of training will be lower as the total cost of training is divided by a larger number of potential participants.

The actual cost-effectiveness of staff training in PBS may be larger than estimated in our conservative calculations, as other participants in the therapist case-load may benefit, particularly those allocated after the trial, or there may have been missing data. It may also be smaller as a result of staff turnover for example. If a therapist’s case-load is 25% larger on average than it was in the trial the total cost of the intervention per participant would be £317. If it is 25% smaller then it would be £529 per participants.

Clinical staff delivering the intervention may not have all been graded at Band 6. Some may be more junior, e.g. a Band 5, at an hourly cost £36 so that and the total cost of delivering the intervention would be £982. If they were more senior (Band 7 at a cost of £52 per hour) the average cost per participant would be £1419.

Combining the upper and lower estimates for training and delivery, the total health and social care service use cost and dividing by proxy reported QALYs at 36 months, the ICER may have a range of £7,422 to £12,897 per QALY gained.

*Supplementary* **References:**

1. Hassiotis A, Poppe M, Strydom A, Vickerstaff V, Hall IS, Crabtree J, et al. Clinical outcomes of staff training in positive behaviour support to reduce challenging behaviour in adults with intellectual disability: cluster randomised controlled trial. The British Journal of Psychiatry. Cambridge University Press; 2018;**212**(3):161–8.
2. Hassiotis A, Poppe M, Strydom A, Vickerstaff V, Hall I, Crabtree J, et al. Positive behaviour support training for staff for treating challenging behaviour in people with intellectual disabilities: a cluster RCT. Health Technol Assess. 2018;**22**(15).
